# Status and factors influencing on-farm conservation of Kam Sweet Rice (*Oryza sativa* L.) genetic resources in southeast Guizhou Province, China

**DOI:** 10.1186/s13002-018-0256-1

**Published:** 2018-11-29

**Authors:** Yanjie Wang, Aixia Jiao, Huicha Chen, Xiaoding Ma, Di Cui, Bing Han, Renchao Ruan, Dayuan Xue, Longzhi Han

**Affiliations:** 1 Institute of Crop Sciences, Chinese Academy of Agricultural Sciences, Beijing, 100081 China; 2 College of Life and Environmental Sciences, Minzu University of China, Beijing, 100081 China; 3 Institute of Crop Germplasm Resources, Guizhou Academy of Agriculture Sciences, Guiyang, 550006 China; 4 Institute of Horticultural Research, Guizhou Academy of Agriculture Sciences, Guiyang, 550006 China

**Keywords:** Kam Sweet Rice (KSR), Genetic resource, On-farm conservation, Ethnic traditional culture, Southeast Guizhou Province

## Abstract

**Background:**

Kam Sweet Rice (KSR) is a special kind of rice landrace that has been cultivated for thousands of years in the borders of Guizhou, Hunan, and Guangxi Provinces of China, and is mainly distributed in southeast Guizhou Province of China currently. KSR has many unique qualities, including strong resistance to diseases, pests, and adverse abiotic conditions, difficulty of threshing, and well glutinous features. KSR germplasm resources are an indispensable material and cultural symbol in the production and daily life and customs of the Dong people. Related studies showed that numerous traditional KSR varieties and cultivation area of KSR decreased sharply from the Qing dynasty to 2015, but many KSR varieties are still conserved in Dong villages of southeast Guizhou Province compared to other areas. However, the number of KSR varieties that are conserved on farms in southeast Guizhou Province and factors influencing the erosion and conservation of KSR genetic resources is unclear. Therefore, this study was an on-farm conservation investigation of KSR genetic resource in China’s major KSR producing areas—Liping, Congjiang, and Rongjiang counties in Guizhou Province and influencing factors analysis of KSR abandonment and conservation.

**Methods:**

The information of KSR conservation status and variety characteristics, typical villages, Dong’s cultural customs, and factors influencing KSR abandonment and conservation was obtained using ethno-biology methods, mainly through field research interviews, including participatory observation, semi-structured interviews, key informant interviews, focus group discussions, and cultural anthropology. The altitude, plant height, awn color and length, hull color, and rice color of 156 KSR accessions in 28 villages were recorded. The variety quantity and cultivation area of KSR were investigated in 33 ethnic villages. Questionnaire surveys were conducted in typical Dong villages to obtain local farmers’ attitudes toward cultivation and protection of KSR. We randomly selected 26 farmers from Sizhai village and 30 farmers from Huanggang village and chose 3 social characteristics including age, gender, and education levels of farmers, and adopted the method of face-to-face interviewing to complete the questionnaires. Then, we analyzed the correlation and determined the significance between farmers with different social characteristics and farmers’ attitudes to KSR development and protection using SPSS 17.0 software.

**Results:**

(1) On-farm conservation status of KSR: a total of 156 KSR varieties were collected from 28 ethnic minority villages from 13 townships (accounting for 21% of three counties) in Liping, Congjiang, and Rongjiang counties. KSR accessions accounted for more than 90% of local rice varieties in each village. According to local farmers, although the quantity of KSR varieties decreased more than 50% in the investigated villages compared to the past 10–20 years, some Dong villages have still cultivated KSR, accounting for more than 50% of the rice field area in 10 villages. This result showed that many KSR varieties are still conserved by in Dong villages, and these KSR varieties have a high genetic diversity of phenotypes. (2) Typical villages investigation: the cultivation area of KSR in Congjiang was the highest, 6.7 times larger than Liping and eight times larger than Rongjiang. In addition, the cultivation area of KSR in Dong villages was larger than that in other ethnic villages, and villages that had a higher planting area of KSR had more KSR accessions. (3) Farmers’ attitude toward the development and conservation of KSR: Dong farmers hold the negative attitudes concerning the development of KSR resources, but they thought it is necessary to protect KSR landraces. Especially, a high level of education and female, young, and old farmers played more important roles in the cultivation and protection of KSR.

**Conclusions:**

Until now, some Dong ethnic villages have still cultivated KSR for thousands of years in Qiandongnan area, although the number of varieties and the planting area of KSR have been greatly reduced. In addition, ethnic traditional culture and social customs were the main influencing factors of KSR conservation; economic, management, and policy factors were the main influencing factors of KSR abandonment. Through the analysis of the correlation between farmers with different social characteristics and their attitudes toward the cultivation, reasons for conservation and abandonment, development tendency, and protection of KSR, we found that a high level of education and female, young, and old farmers play more important role in the cultivation and protection of KSR. Therefore, in order to promote the protection and sustainable utilization of KSR, it is necessary to build on-farm conservation of KSR and improve the position of female farmers and the education level of young people, and encourage the old people to educate the middle-aged to conserve and protect KSR as well as Dong’s traditional culture and social customs. This study is of great significance to promote better protection and optimal utilization of KSR and enable the public, government, and related researchers pay more attention to conserving ethnic traditional cultures.

## Background

Kam Sweet Rice (hereinafter referred to as KSR) is a traditional rice variety that originated from the complicated ecological environment at the borders of Guizhou, Hunan, and Guangxi Provinces, over a long period of natural evolution and artificial selection under the Dong minority traditional farming system. KSR is not a biological taxonomic unit, but is an original, ecological rice landrace [1]. KSR is only distributed within the borders of Guizhou, Hunan, and Guangxi Provinces and is mainly cultivated in forest ravines in Qiandongnan Autonomous Prefecture of Guizhou Province. KSR is highly adapted to the local climate and ecological environment. The cultivation of KSR resources in Qiandongnan has a very long history, dating back more than 2000 years [2]. Qiandongnan is one of the birthplaces of a “glutinous-food culture”, where the Dong, Miao, Shui, Zhuang, and other ethnic groups live together. KSR is more prominent in Dong culture and is fully embedded in daily production and life, national customs, and religious beliefs of the Dong people [3]. The main cultivators of KSR are the Dong people (also called the Kam people). Kam is one of 56 ethnic groups officially recognized by the People’s Republic of China. They are famed for their native-bred KSR, carpentry skills, and unique architecture, in particular a form of covered bridge known as a “wind and rain bridge” and a high tower known as the “Drum-tower” [4].

The Dong people separate local rice varieties into two types, He and Gu. The Gu type is easy to thresh, whereas the He variety is extremely difficult to thresh in the natural environment and requires artificial harvesting with a traditional pick tool, known as “grain knife” [5]. The He type has many different accessions, including *indica* and *japonica*, early, middle, and late maturing, non-glutinous and glutinous, and can also have black, red, or white episperm. Currently, most KSR cultivated in paddy fields in China is *japonica*, middle and late maturing, glutinous, white rice [6]. Among He rice germplasm resources, more than 90% are the glutinous type, and the best quality He rice is KSR, due to its strong aromatic flavor [7]. KSR was first reported in Science in 2008 [8], and the Food and Agriculture Organization (FAO) called it a “Worldwide Specialty Rice” [9].

In the 1980s, a total of 332 varieties of KSR were collected in the main distribution area—Qiandongnan Prefecture Liping, Congjiang, and Rongjiang counties—and stored in the China National Gene Bank. However, high-yielding rice varieties such as short-stalk rice and hybrid rice have been promoted by the Chinese government since the 1980s, and the government made a policy that required switching to *indica* rice. This policy caused the planting area and the number of varieties of KSR to sharply decrease. According to a study of local agricultural history experts, Tianzhu, Jinping, and Jianhe counties near the Guizhou Qingshui River and Liping, Congjiang, and Rongjiang Counties near the Duliu River were still the main growing area of KSR until the late Qing dynasty [10], and there were as many as hundreds of thousands of varieties. However, only a small number of current Dong villages in Liping, Congjiang, and Rongjiang have preserved KSR genetic resources. From Qing dynasty to 2010, numerous traditional KSR landraces have been lost, the proportion of KSR planting areas decreased from 60 to 7% in southeast Guizhou, and by the end of 2015, the 363 landraces investigated in the early 1980s in Congjiang, Liping, and Rongjiang counties had decreased by 72.5% [11].

KSR varieties still retain some characteristics of wild rice. Compared with other rice landraces from Guizhou Province, KSR has some unique features: it is taller; has thicker and wider leaves, a thicker stem, larger spike, and more awns; but is intolerant to fertilizer and is susceptible to lodging. Furthermore, KSR can only be planted in certain areas; it is highly susceptible to changes in temperature and light and has a long growth period and late maturity. However, KSR has resistance to cold, drought, flood, and shade and shows excellent characteristics that are suitable for growth in mountainous areas [12]. In recent years, studies on KSR have focused on photothermal response characteristics [13], rice quality [14], wide compatibility with other rice varieties [15], hybridization breeding [16], variety production test [17], identification of cold resistance and drought resistance [18], genetic diversity analysis [19, 20], collection and preservation of germplasm resources [21], naming system of the Dong people [22], planting history from the perspective of social and cultural changes [10], and the relationship with ethnic traditional culture [21, 23]. In addition, concentration of Abscisic Acid (ABA) and the volatile components of cooked Gou Cengao (an indigenous aromatic KSR variety of Congjiang County) and rice grains from the filling stage were analyzed to reveal the correlation between ABA and key volatile compounds [24]. However, on-farm conservation status of KSR and factors influencing the conservation and abandonment by Dong villages in recent year is unclear, and thus, studies are required to promote the protection and continued use of KSR.

Rice landraces evolved through many generations and are suited to the local conditions of farms. These landraces reflect socio-cultural preferences and are identified by various vernacular names. India is home to many rice landraces, and the ones from the state of West Bengal and the north eastern states are especially diverse morphologically and genetically [25]. Indian researchers have conducted many studies on collection and conservation of rice landraces. Rana et al. [26] collected rice genetic resources for about 8 years (1999–2006), and 1069 germplasm accessions including 154 named landraces were collected in the western Himalayan region of India. Mathure et al. [27] collected 88 aromatic rice cultivars from Maharashtra State and assessed the determinants of kernel quality and grain morphology. Baharul et al. [28] found that traditionally cultivated indigenous rice varieties in northeast India show high levels of genetic diversity compared to levels of genetic diversity reported from wild rice populations in various parts of the world. These rice landraces have withstood biotic and abiotic stresses, are suited to the local conditions of farms, reflect socio-cultural preferences, and can still be found in crop fields located distantly in rural and tribal areas [29]. Rice landraces are a major means of survival for marginal and impoverished tribal farmers in biodiversity hotspots, including southwest China, western Himalaya. On-farm conservation of rice landraces is also a means of protection of culture, heritage, and socio-economic structure.

On-farm conservation of crop genetic resources is a prerequisite for sustainable food production because this practice not only conserves the genetic resources of crops and evolutionary processes that involve diversity, but also the traditional knowledge system [30]. Many studies have focused on on-farm conservation for different crop varieties recently, including studies of vegetable landraces in the Korça region [31], a lentil landrace of Zaer [32], horticultural crops and wild fruit species in central Asia [33], and indigenous fruit species in Nigeria [34]. On-farm conservation can be enhanced by improving farmers’ perceptions [35]. It is crucial to obtain farmers’ opinions about whether they are willing to cultivate traditional varieties, the perceived advantages and disadvantages of the traditional varieties, development tendencies, and whether it is necessary for crop landraces to be protected. When a farmer chose a new variety to replace a traditional one, it reflected the farmer’s judgment that the new variety offers benefits or advantages [36]. Wale [37] found that farmers believed that the decrease of traditional varieties was due to low yield and land unsuitability. Jani [30] found that cultural identity, traditional production techniques, the level of self-sufficiency, and organoleptic qualities are the main factors determining the conservation of traditional cultivars. Therefore, understanding the perception of farmers about conservation of KSR biodiversity is an effective strategy for sustainable adoption and use of KSR genetic resources and enhances the comparative advantage of the landraces. This study therefore assessed farmers’ perception toward the cultivation and protection of local KSR.

KSR has a long history of cultivation and unique quality characteristics, and it is used in a special way by local ethnic minorities, which has determined its important position in the history of Dong rice culture in China. Although a large number of KSR have disappeared, many KSR varieties are still conserved in Dong villages of southeast Guizhou Province compared to other areas. However, the number of KSR varieties that are conserved on farms in southeast Guizhou Province and factors influencing the erosion and conservation of KSR genetic resources is currently unclear. Therefore, we selected the main production regions—28 ethnic villages from 13 towns in Liping, Congjiang, and Rongjiang counties of Qiandongnan Autonomous Prefecture, and collected KSR varieties conserved by local farmers to understand the on-farm conservation status of KSR and biological characteristics related to the conservation of these varieties. We also investigated 33 ethnic villages from 15 towns to get information about the cultivation area, number of accessions, and cultivation history of KSR in different villages. In addition, we selected two Dong villages, surveyed farmers of different gender, age, and cultures to determine the factors influence preserving or abandoning KSR, and analyzed the influencing factors from the aspects of ethnic culture, social customs, economic benefits, and environmental conditions in detail. This study is of great significance to better protect and optimize the use of KSR resources and will provide guidance for protecting and retaining KSR genetic resources and Dong’s traditional culture and social customs.

## Methods

### Study site

**Fig. 1:**
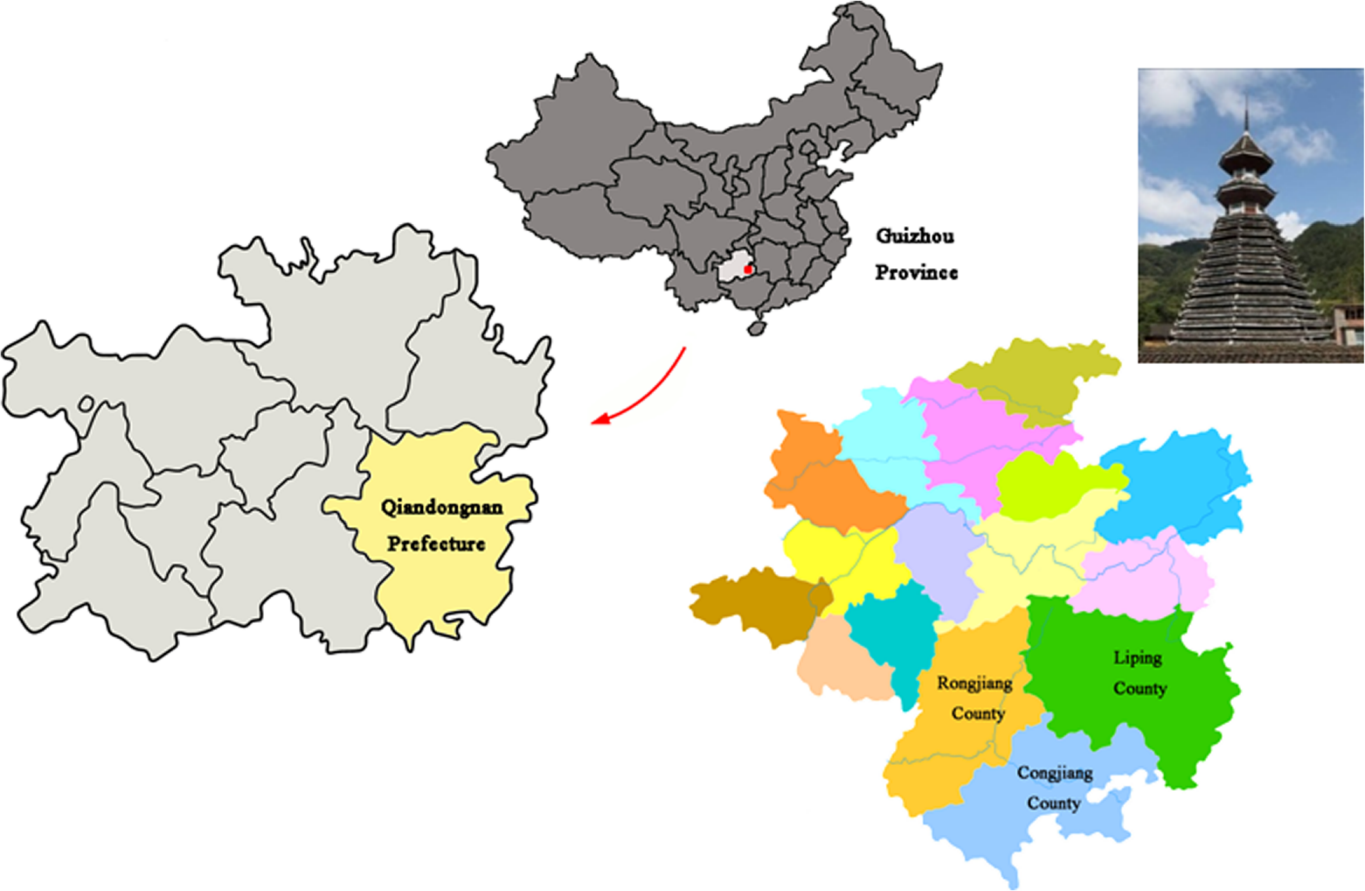
The main production areas of KSR

Liping, Congjiang, and Rongjiang counties (E 108° 04′ to 109° 31′, N 25° 16′ to 26° 28′), the main production areas of KSR, are part of Qiandongnan Miao and Dong Autonomous Prefecture, located in the southeast part of Guizhou Province in the People’s Republic of China, bordering Hunan to the east and Guangxi to the south. Qiandongnan has an area of 30,339 km^2^ (Fig. 1). The altitude in this region varies greatly from 137 to 2187 m, and the area has a complex terrain of 90% mountains, 5% water, and 5% farmland. The average annual rainfall is 1200 mm to 1400 mm. The large number of genetic resources of traditional crops is due to the wide geographic and climatic distribution in this region. These three counties are not only the origin of traditional Dong culture but also the source of KSR genetic resources.

### KSR collection and field investigation

The information of KSR conservation status and variety characteristics, typical villages, Dong’s cultural customs, and factors influencing KSR abandonment and conservation were obtained using ethno-biology methods, mainly through field research interviews, including participatory observation, semi-structured interviews, key informant interviews, focus group discussions, and cultural anthropology [38, 39]. From October to November in 2013 and 2015, KSR was collected in the field in 28 ethnic villages distributed in Liping, Congjiang, and Rongjiang counties of Guizhou. In the sampled villages, almost every farmer had KSR; therefore, we usually asked the village head or elder to help us collect KSR varieties, because they were more familiar with the situation in their villages. The altitude, plant height, awn color and length, hull color, and rice color of KSR were recorded. We selected 33 typical villages to investigate the number of KSR varieties, and the cultivation area of KSR (including 28 on-the-spot investigated villages and the other 5 not on-the-spot investigated villages). The selected villages cover an altitude gradient above sea level from 240 to 935 m, have a range of agro-ecological conditions, and are inhabited by different minority ethnic groups, mainly Dong, but also Miao, Yao, Zhuang, and Shui people.

### Questionnaire survey

Questionnaire surveys were conducted in typical Dong villages to obtain local farmers’ attitudes toward cultivation and protection of KSR. We randomly selected 26 farmers from Sizhai village and 30 farmers from Huanggang village (both are Dong villages). The interviewees were all Dong people, and we chose 3 social characteristics including age, gender, and education levels of different farmers and adopted the method of face-to-face interviewing to complete the questionnaires. Then, we analyzed the correlation and significant difference between farmers’ attitudes to KSR development and protection and farmers with different social characteristics using SPSS 17.0 software. In this study, 56 questionnaires were issued and 56 valid questionnaires were collected, the effective rate was 100%. The questionnaire is shown in Table 1.

**Table 1:** Questionnaire of farmers’ attitude to the development and protection of KSR

	Questions	Answers	
Social characteristics	Q1: Gender	A: Male	B: Female
	Q2: Age	A: ≤ 20 years C: 36–60 years	B: 21–35 years D: ≥ 61 years
	Q3: Education level	A: Illiterate C: Middle school	B: Primary school D: High school or above
Farmers’ attitudes concerning the development and influencing factors of KSR	Q4: Will you continue to cultivate KSR? (Choose the next question depending on your answer)	A: Certainly, I will cultivate KSR for hundreds or thousands of years B: I will cultivate KSR in my life, but uncertain if descendants will cultivate C: I will cultivate KSR for several years D: I will not cultivate KSR	
	Q5: Why do you want to continue to cultivate KSR?	A: Food culture, festival celebrations, belief sacrifice (Culture factor) B: Taste habits, gift; Rice-Fish-Duck Symbiotic System (Social factor) C: High market price and make many by-products (Economic factor) D: Suitable to local climate environment, disease and pest resistance (Environment factor)	
	Q6: Why don’t you want to continue to cultivate KSR?	A: Dong’s traditional festivals are fading away and the demand of KSR is greatly reduced (Culture factor) B: The taste of new variety is better than the old KSR; not enough labor because of outside employment (Social factor) C: Low yield, time and labor consuming, government policy (Economic, management, and policy factor) D: Unsuitable to local climate condition, disease and pest resistance disappearing (Environment factor)	
	Q7: What’s your opinion of KSR development tendency?	A: Less and less C: More and more	B: Maintain the status quo D: Unknown
	Q8: What’s your opinion of taking effective measures to protect KSR?	A: Necessary	B: Unnecessary

## Results

### On farm conservation status of KSR in Qiandongnan

**Fig. 2:**
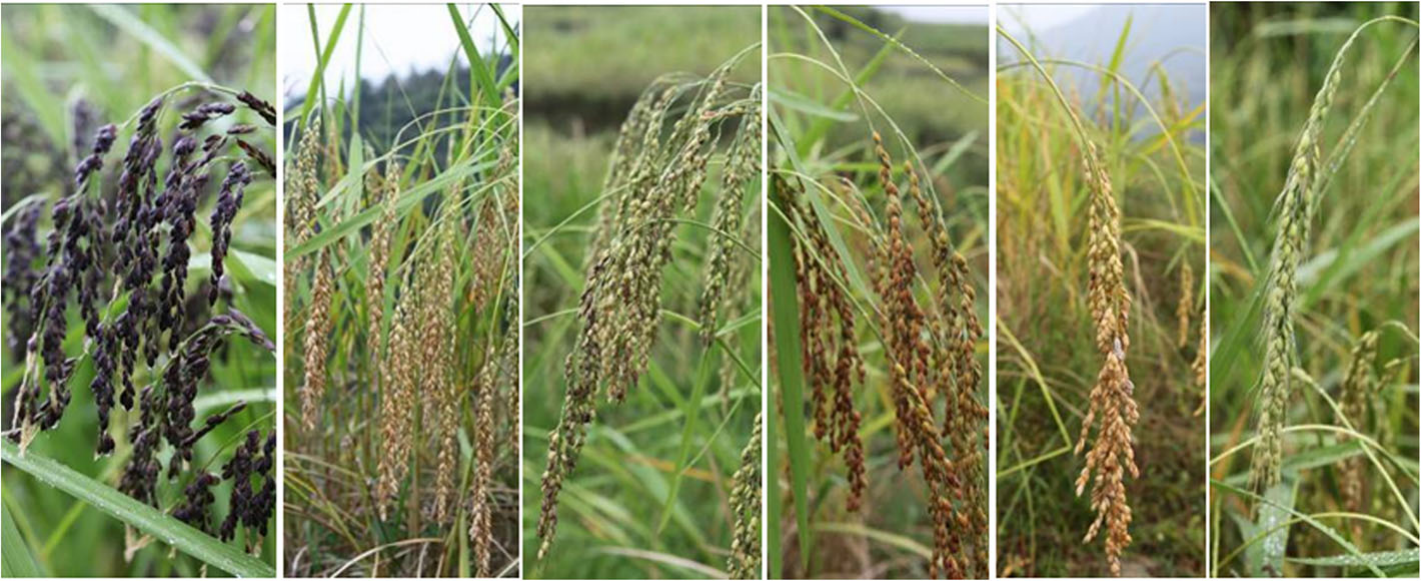
Examples of KSR varieties conserved in Dong villages

A total of 156 KSR varieties were collected from 28 ethnic minority villages from 13 townships (accounting for 21% of three counties) in Liping, Congjiang, and Rongjiang counties (Table 2). KSR accessions accounted for more than 90% of local rice varieties in each village. According to local farmers, although the quantity of KSR varieties decreased more than 50% in the investigated villages compared to the past 10–20 years, some Dong villages have still cultivated KSR, accounting for more than 50% of the rice field area in 10 villages. This result showed that for ethnic villages, especially in Dong villages, many KSR varieties were still conserved by local farmers. Dong, Miao, Yao, and Zhuang ethnic groups bred various KSR varieties suitable for local ecological environments and planting altitudes. Because of this, the plant height, awn length, awn color, glume color, and rice color were different among varieties; each variety has outstanding characteristics, and there is high genetic diversity of phenotypes (Fig. 2).

**Table 2:** Kam Sweet Rice varieties collected in Liping, Congjiang, and Rongjiang counties of Guizhou Province

No.	Landraces	Altitude (m)	Plant height (cm)	Awn color	Awn length	Hull color	Rice color	Special characteristic	Location Village/town/County
1	Wuminghe	500	125	Red	Medium	Russet	White	Wide altitude adaption	Yandong, Yandong, Liping
2	Baixianghe	500	130	Yellow	Short	Yellow	White	Strong aromatic flavor and large area	Yandong, Yandong, Liping
3	Huangshanxue	500	125	Black	Long	Purplish black	Black	Medicinal value, no pest and disease	Yandong, Yandong, Liping
4	Niumanghe	400	125	Red	Medium	Yellow	White	Strong waxy, < 500 m elevation	Xiaozhai, Yandong, Liping
5	Rongzhuhe	400	135	None		Yellow	White	Curd rice, suitable to cold paddy field	Xiaozhai, Yandong, Liping
6	Guiyanghe	400	110	Black	Short	Yellow	White	Wide altitude adaption, suitable to cold paddy field	Xiaozhai, Yandong, Liping
7	Dongsuihe	335	130	None		Yellow	White	Adapt to the elevation below 500 m	Zaigong, Yandong, Liping
8	Ronghe	335	110	Red	Medium	Yellow	White	Strong root, lodging-resistant	Zaigong, Yandong, Liping
9	Honghe1	735	120	Red	Short	Russet	White	Strong cold-resistant, area less than Liezhuhe of Huanggang	Huanggang, Shuangjiang, Liping
10	Honghe2	735	130	Red	Medium	Russet	White	Longer awn than Honghe1	Huanggang, Shuangjiang, Liping
11	Liezhuhe	735	125	Red	Short	Yellow	White	Well-adapted & the largest area in Huanggang	Huanggang, Shuangjiang, Liping
12	Old-Liezhuhe	735	140	Red	Medium	Yellow	White	No lodging-resistant, Strong cold-resistant	Huanggang, Shuangjiang, Liping
13	60-days He	735	110	Black	Medium	Yellow	White	Strong drought resistant	Huanggang, Shuangjiang, Liping
14	70-days He	735	120	None		Yellow	White	Strong drought resistant	Huanggang, Shuangjiang, Liping
15	Jindongnuo	735	110	White	Medium	Yellow	White	Low yield, rarely cultivated	Huanggang, Shuangjiang, Liping
16	Yangnong	735	125	White	Long	Yellow	White	Suitable to cold and short solar environment	Huanggang, Shuangjiang, Liping
17	Bianlongtu	735	125	Red	Medium	Yellow	White	Introduced from Longtu town Congjiang County	Huanggang, Shuangjiang, Liping
18	Baimangwanshunuo	735	135	White	Short	Yellow	White	High yield	Huanggang, Shuangjiang, Liping
19	Heimangwanshunuo	735	135	Black	Short	Yellow	White	High yield	Huanggang, Shuangjiang, Liping
20	Gouli	735	125	Red	Medium	Yellow	White		Huanggang, Shuangjiang, Liping
21	Yehe	735	130	Black	Short	Yellow	White		Huanggang, Shuangjiang, Liping
22	Dewuhe	735	129	Black	Short	Yellow	White		Huanggang, Shuangjiang, Liping
23	Shudonghe	735	132	None		Yellow	White		Huanggang, Shuangjiang, Liping
24	Banpohe	735	130	Brown	Medium	Yellow	White		Huanggang, Shuangjiang, Liping
25	Gaojinghe	735	129	None		Yellow	White		Huanggang, Shuangjiang, Liping
26	Liezhuhe	380	126	Red	Medium	Yellow	White	Strong aromatic flavor	Huanggang, Shuangjiang, Liping
27	Goubingman	735	104	Yellow	Long	Yellow	White		Huanggang, Shuangjiang, Liping
28	Shanshupi	735	131	Red	Medium	Russet	White		Huanggang, Shuangjiang, Liping
29	Heihe	735	106	Black	Short	Purplish black	White	Strong aromatic flavor	Huanggang, Shuangjiang, Liping
30	Heimanghe	380	150	Black	Very long	Purplish black	White	Very late-maturing variety	Kengdong, Shuangjiang, Liping
31	Niumaohe	380	160	Black	Medium	Yellow	White	No lodging-resistant	Kengdong, Shuangjiang, Liping
32	Rongdonghe	380	135	Red	Long	Russet	Red	No strong waxy	Kengdong, Shuangjiang, Liping
33	Gonggenghe	380	125	Black	Long	Yellow	White	Strong aromatic flavor, the largest area in Kengdong	Kengdong, Shuangjiang, Liping
34	Danuo	380	95	Red	Short	Yellow	White	Lodging-resistant	Kengdong, Shuangjiang, Liping
35	Heinuo	380	150	None		Yellow	Black	No lodging-resistant	Kengdong, Shuangjiang, Liping
36	Gouzaige1	380	145	White	Long	Yellow	White	Wide adaption range, more aromatic than Baixianghe	Kengdong, Shuangjiang, Liping
37	Rongtanghe	380	103	Yellow	Medium	Yellow	White	Strong aromatic flavor, adapt to medium elevation	Kengdong, Shuangjiang, Liping
38	Tonghe	380	119	None		Yellow	White	No barren-resistant	Kengdong, Shuangjiang, Liping
39	Heihe	380	124	Purple	Short	Brown	Red		Kengdong, Shuangjiang, Liping
40	Gouzaige2	448	105	Yellow	Medium	Yellow	White	Wide adaption, strong aromatic flavor, easy lodging	Miedong, Shuangjiang, Liping
41	Ougen	448	101	Yellow	Medium	Yellow	White	Strong aromatic flavor	Miedong, Shuangjiang, Liping
42	Hongmangbainuo	458	120	Red	Long	Yellow	White	Strong disease-resistant	Zhaoxing, Zhaoxing, Liping
43	Changmangdanuo	424	120	White	Long	Yellow	Red	Little waxy, rice harder than black he	Zhaoxing, Zhaoxing, Liping
44	Heinuo	458	130	None		Purplish black	Black	Adapt to cold water, strong disease-resistant	Zhaoxing, Zhaoxing, Liping
45	Qiyuenuo	351	120	None		Yellow	White	Short growth period	Zhaoxing, Zhaoxing, Liping
46	Youmangdanuo	424	130	White	Medium	Yellow	White	Barren-resistant	Zhaoxing, Zhaoxing, Liping
47	Hongpibainuo	350	110	Red	Medium	Russet	White	Lodging-resistant	Zhaoxing, Zhaoxing, Liping
48	Deshunbayuehe	420	140	None		Yellow	White	Good materials for oil tea	Deshun, Deshun, Liping
49	Deshunnuohe	420	110	White	Medium	Yellow	Red	Introduced from Guangxi, cultivated 30 years	Deshun, Deshun, Liping
50	Bayuehe	520	130	Black	Short	Russet	White	Good materials for Dongguo	Pingfu, Deshun, Liping
51	Xibaihe	922	120	White	Long	Yellow	White	Aromatic flavor, good quality than Zhongbaihe	Jiaodong, Shangzhong, Liping
52	Zhongbaihe	922	120	White	Medium	Yellow	White	Adapt to medium-low fertilizer soil	Jiaodong, Shangzhong, Liping
53	Dabaihe	922	120	White	Medium	Yellow	White	Waxy and aromatic flavor less than Xibaihe	Jiaodong, Shangzhong, Liping
54	Jidenghonghe	593	115	White	Long	Yellow	Red	Strong pest and disease resistant	Jideng, Shangzhong, Liping
55	Shuiniumao	596	130	Black	Verylong	Yellow	Red	Special and appreciable variety, wide adaption	Cengguan, Leidong, Liping
56	Wumaohe	596	125	Black	Short	Yellow	White	Rice harder than Shuiniumao	Cengguan, Leidong, Liping
57	Gouduwei	250	120	Red	Medium	Yellow	White	Late-maturing, wide adaption range	Leidong, Leidong, Liping
58	Goudusheng	250	110	Black	Short	Yellow	White	Early-maturing, wide adaption range	Leidong, Leidong, Liping
59	Rongjiangxianghe	576	112	Yellow	Short	Yellow	White	Early-maturing	Zaima, Zaima, Rongjiang
60	Yangwenna	618	109	Purple	Short	Yellow brown	White		Xiaohuang, Gaozeng, Congjiang
61	Lijiuhe	618	110	None		Yellow	White		Xiaohuang, Gaozeng, Congjiang
62	Dabaohe	618	105	Purple	Verylong	Yellow	Red	No aromatic flavor	Xiaohuang, Gaozeng, Congjiang
63	Yangwan	618	104	Purple	Short	Yellow	White	No aromatic flavor	Xiaohuang, Gaozeng, Congjiang
64	Gouhagongniu	618	102	Red	Long	Russet	White	Strong aromatic flavor	Xiaohuang, Gaozeng, Congjiang
65	Outang	618	109	Yellow	Short	Yellow	White	Strong aromatic flavor	Xiaohuang, Gaozeng, Congjiang
66	Heihe	277	114	Purple	Short	Purplish black	Red		Yinping, Gangbian, Congjiang
67	Yinpingnuohe	277	116	Red	Short	Russet	White		Yinping, Gangbian, Congjiang
68	Huangkenuohe	277	124	Yellow	Short	Yellow	White		Yinping, Gangbian, Congjiang
69	Heimangnuohe	277	109	Black	Short	Yellow	White		Yinping, Gangbian, Congjiang
70	Xianghenuo	277	106	Purple	Short	Yellow	White		Yinping, Gangbian, Congjiang
71	Yinpingxianghe1	277	117	Purple	Short	Yellow	White		Yinping, Gangbian, Congjiang
72	Yinpingxianghe2	277	137	Yellow	Short	Yellow	White		Yinping, Gangbian, Congjiang
73	Goucengao	600	100	Brown	Short	Yellow	White	High elevation, stable character, cold-resistant	Biapa, Gaozeng, Congjiang
74	Xianghenuo	600	103	Brown	Short	Yellow	White		Biapa, Gaozeng, Congjiang
75	Biapanuohe	600	107	Purple	Short	Yellow	White		Biapa, Gaozeng, Congjiang
76	Xianghenuo	600	106	Red	Short	Yellow	White		Biapa, Gaozeng, Congjiang
77	Baixianghe	582	102	Purple	Short	Purplish black	White		Xinsheng, Gaozeng, Congjiang
78	Baixianghe	582	100	Purple	Short	Purplish black	White		Xinsheng, Gaozeng, Congjiang
79	120-days he	582	113	Brown	Short	Yellow brown	White	Early-maturing, no aromatic flavor, drought-enduring	Xinsheng, Gaozeng, Congjiang
80	Gouyangdang	582	102	Yellow	Medium	Yellow	White	Early-maturing, aromatic flavor, high elevation	Xinsheng, Gaozeng, Congjiang
81	Gounong	582	111	Purple	Short	Purplish black	Black	Black glutinous rice, medical value, low yield	Xinsheng, Gaozeng, Congjiang
82	Xinshengnuohe1	582	104	Brown	Short	Yellow	White		Xinsheng, Gaozeng, Congjiang
83	Xinshengnuohe 2	582	106	Yellow	Short	Yellow	White		Xinsheng, Gaozeng, Congjiang
84	Biashaxianghe1	550	100	Red	Short	Russet	White		Biasha, Bingmei, Congjiang
85	Biashaxianghe 2	550	121	Purple	Short	Yellow	White		Biasha, Bingmei, Congjiang
86	Biashaxianghe 3	550	98	Red	Short	Yellow	White		Biasha, Bingmei, Congjiang
87	Goudaha	380	96	Red	Short	Russet	White	Strong aromatic flavor	Zhanli, Gaozeng, Congjiang
88	Heinuo	380	113	Purple	Short	Purplish black	White	No aromatic flavor	Zhanli,Gaozeng,Congjiang
89	Gouliezhu	380	122	Yellow	Short	Yellow	White		Zhanli, Gaozeng, Congjiang
90	Dabaohe	380	103	Red	Long	Red	White	Strong aromatic flavor	Zhanli, Gaozeng, Congjiang
91	Dudui	380	111	Yellow	Long	Yellow	White	The most aromatic, low yield	Zhanli, Gaozeng, Congjiang
92	Liangliang	380	137	Brown	Long	Brown	White		Zhanli, Gaozeng, Congjiang
93	Bianman	380	110	Yellow	Long	Yellow	White	Strong aromatic flavor	Zhanli, Gaozeng, Congjiang
94	Ganyuan	380	112	Brown	Short	Yellow	White	Strong aromatic flavor	Zhanli, Gaozeng, Congjiang
95	Goudong	380	119	Yellow	Short	Yellow	White		Zhanli, Gaozeng, Congjiang
96	Ronglan	380	139	Yellow	Verylong	Yellow	Red	No aromatic flavor	Zhanli, Gaozeng, Congjiang
97	Gonggu	380	106	Red	Long	Russet	White	Full seed	Zhanli, Gaozeng, Congjiang
98	Gounen	380	134	Black	Short	Purplish black	White		Zhanli, Gaozeng, Congjiang
99	Yangdanghe	380	125	Yellow	Medium	Yellow	White	Strong aromatic flavor	Zhanli, Gaozeng, Congjiang
100	Rongdonghe	380	125	None		Yellow	White		Zhanli, Gaozeng, Congjiang
101	Heipihe	380	137	Black	Short	Purplish black	White		Zhanli, Gaozeng, Congjiang
102	90-days he	380	125	None芒		Yellow	White		Zhanli, Gaozeng, Congjiang
103	Wangni	380	141	Yellow	Short	Yellow	White		Zhanli, Gaozeng, Congjiang
104	Yansanse	380	125	Red	Short	Red	White	Strong aromatic flavor	Zhanli, Gaozeng, Congjiang
105	Huangchuan	380	135	Yellow	Short	Yellow	White		Zhanli, Gaozeng, Congjiang
106	Bazhou red he	380	128	Red	Verylong	Red	White		Zhanli, Gaozeng, Congjiang
107	Goutun	380	131	Yellow	Medium	Yellow	White		Zhanli, Gaozeng, Congjiang
108	Tumanghe	380	131	Yellow	Medium	Yellow	White		Zhanli, Gaozeng, Congjiang
109	Xiushuihe	380	132	Yellow	Long	Yellow	White		Zhanli, Gaozeng, Congjiang
110	Bazhou black he	380	121	None		Brown	Black		Zhanli, Gaozeng, Congjiang
111	Diping black he	380	161	None		Purplish black	Black		Zhanli, Gaozeng, Congjiang
112	White xian he	380	160	Yellow	Verylong	Yellow	White		Zhanli, Gaozeng, Congjiang
113	Kengdonghonghe	380	158	Red	Medium	Red	White	Light in aromatic flavor	Zhanli, Gaozeng, Congjiang
114	Zhanlinuohe1	380	114	Yellow	Short	Yellow	White	No aromatic flavor	Zhanli, Gaozeng, Congjiang
115	Zhanlinuohe2	380	109	Yellow	Long	Yellow	White	Very strong aromatic flavor	Zhanli, Gaozeng, Congjiang
116	Biapagonghe	600	113	Black	Medium	Yellow	White	Low elevation, mixed cultivation with Biapa female he	Biapa, Gaozeng, Congjiang
117	Biapamuhe	600	121	Black	Medium	Purplish black	Red	Low elevation, mixed cultivation with Biapa female he	Biapa, Gaozeng, Congjiang
118	Danianhe	600	105	Black	Short	Yellow	White	No aromatic flavor	Biapa, Gaozeng, Congjiang
119	Jiuyuejiu	600	119	Yellow	Medium	Yellow	White	Strong aromatic flavor	Biapa, Gaozeng, Congjiang
120	Gouhadang	600	102	Red	Long	Russet	White	Strong aromatic flavor	Biapa, Gaozeng, Congjiang
121	Gougong	600	109	Red	Medium	Yellow	White	No aromatic flavor	Biapa, Gaozeng, Congjiang
122	Gouliedainian	800	111	Purple	Short	Yellow	White	No aromatic flavor	Jianhua, Gaozeng, Congjiang
123	Gourongtun	800	108	Yellow	Short	Yellow	White	High yield, late-maturing, no aromatic flavor	Jianhua, Gaozeng, Congjiang
124	Gougaoqian	800	112	Yellow	Short	Yellow	White	Strong aromatic flavor	Jianhua, Gaozeng, Congjiang
125	Goubaisan	800	119	None		Yellow	White	No aromatic flavor	Jianhua, Gaozeng, Congjiang
126	Gouhuanggang	800	111	Brown	Short	Russet	White	Introduced from Huanggang village, strong aromatic flavor	Jianhua, Gaozeng, Congjiang
127	Goucengaoka	800	103	Red	Very long	Russet	White	Aromatic flavor	Jianhua, Gaozeng, Congjiang
128	Gouliejiu1	800	112	None		Yellow	White	Early-maturing, no aromatic flavor, low elevation	Jianhua, Gaozeng, Congjiang
129	Gouliejiu2	800	115	Red	Short	Yellow	White	Late-maturing, no aromatic flavor, low elevation	Jianhua, Gaozeng, Congjiang
130	130-days He	800	118	None		Yellow	White	Drought-resistant, aromatic flavor, wide adaption	Jianhua, Gaozeng, Congjiang
131	Jianhuanuohe	800	136	Black	Short	Yellow	White		Jianhua, Gaozeng, Congjiang
132	Gouyue	430	119	None		Yellow	White	Stabilization, desease-resistant, large area, long history	Longjiang, Yongli, Congjiang
133	Goudai	430	113	Brown	Long	Yellow brown	White	Strong aromatic flavor, short growth period	Longjiang, Yongli, Congjiang
134	Goudaihuo	430		Red	Medium	Yellow brown	White	Aromatic flavor, short stalk, easy lodging in fertilizer soil	Longjiang, Yongli, Congjiang
135	Goudong(big spike)	430	141	Yellow	Short	Yellow	White	Big spike, high yield, no aromatic flavor	Longjiang, Yongli, Congjiang
136	Goudong(red rice)	430	117	None		灰yellow	Red	No aromatic flavor, low yield	Longjiang, Yongli, Congjiang
137	Darongnuo	430	114	None		Yellow	White	Introduced from Darong village	Longjiang, Yongli, Congjiang
138	Gouyongmi	240	111	Yellow	Short	Yellow	White	Wide adaption range, more tillers, no aromatic flavor	Dingdong, Xishan, Congjiang
139	Gouyongwai	240	114	Yellow	Long	Yellow	White	Very aromatic and waxy, good taste, long history	Dingdong, Xishan, Congjiang
140	Dingdongheihe	240		Yellow	Long	Brown	Black	Wide adaption range	Dingdong, Xishan, Congjiang
141	Goudainian	240	128	None		Yellow brown	White	The most aromatic, soft and waxy, good taste	Dingdong, Xishan, Congjiang
142	Goudang	240	100	Red	Long	Red	White	Strong aromatic flavor	Dingdong, Xishan, Congjiang
143	Biaogei	544	125	Yellow	Short	Purplish black	Black	Low yield, resistant to disease & pest	Baiweng, Cuili, Congjiang
144	Biaodang	544	101	Red	Long	Red	White	Strong aromatic flavor, low elevation	Baiweng, Cuili, Congjiang
145	Biaomiu	544	115	Purple	Short	Purplish black	White	Introduced from Gugangxi 10 years ago	Baiweng, Cuili, Congjiang
146	Biaobu	544	117	None		Yellow	White	High yield, big panicle & thick stalk, no aromatic flavor	Baiweng, Cuili, Congjiang
147	Dongbiao	544	121	None		Yellow	White	Strong aromatic flavor, high yield, large area	Baiweng, Cuili, Congjiang
148	Dainianya	500	124	Black	Short	Yellow	Red	Red rice, low elevation of 400 m	Zaizhuan, Cuili, Congjiang
149	Heikehe	500	124	Black	Long	Purplish black	Red	Low yield, black hull & red rice	Zaizhuan, Cuili, Congjiang
150	Goudang	500	105	Red	Short	Russet	White	Strong aromatic flavor, Adapt to a wide range of elevation	Zaizhuan, Cuili, Congjiang
151	Goudaian1	500	120	Purple	Medium	Yellow brown	White	Strong disease-resistant, piebald hull	Zaizhuan, Cuili, Congjiang
152	Goudaian2	500	118	Purple	Short	Yellow	White	Strong aromatic flavor, yellow hull	Zaizhuan, Cuili, Congjiang
153	Gounong	470	129	Black	Medium	Purplish black	Red	Black hull & red rice, low yield	Gaowen, Cuili, Congjiang
154	Houyanghaogun	470	100	Purple	Short	Yellow	White	Short awn, no aromatic flavor	Gaowen, Cuili, Congjiang
155	Goudaian3	470	112	Yellow	Short	Yellow	White	Awn, large area	Gaowen, Cuili, Congjiang
156	Goudaian4	470	116	None		Yellow	White	No awn, Strong aromatic flavor	Gaowen, Cuili, Congjiang

KSR was generally suitable for planting in yellow soil at altitudes below 1000 m, but different varieties had different optimal altitudes. For elevations less than 700 m, 79% of KSR rice varieties were suitable for planting, but KSR from Huanggang and Jiaodong villages in Liping County and Jianhua village in Congjiang County were suitable for high altitudes above 700 m. In addition, three varieties—Wuminghe, Liezhuhe, and Goucengao—cultivated in high and low altitudes. Wuminghe was the main variety in Yandong, Kengdong, and Miedong villages. Liezhuhe and Goucengao were the largest varieties of planting area in Huanggang and Jianhua villages, respectively.

A total of 84% of KSR varieties were awny and had different length and color. For awn color overall, the amount with yellow or red awn was equal, accounting for 53% of the total; black and purple accounted for 18% and 14% of the total, respectively; and white and brown accounted for 8% and 7% of the total, respectively. For awn length, most KSR varieties had a short awn (less than or equal to 1 cm), accounting for half of the total; the second most common was a medium-sized awn (1–3 cm), accounting for 26% of the total; a long awn (3–5 cm) accounted for 19% of the total; and an extremely long awn (> 5 cm) accounted for 5% of the total. For glume colors of KSR, 72.4% were yellow, yellow brown, or gray yellow; 10.9% of varieties were purple black; and 16.7% were red, brown, or red brown. For seed color, 86% of KSR were white, 9% were red, and 8 varieties—Huangshanxue, Heinuo, Zhaoxingheinuo, Gounong, Bazhouheihe, Dipingheihe, Dingdongheihe, and Biaoji—were black. Most KSR had long stalks: 70% were 111–161 cm, of which 9 cultivars were between 141 and 161 cm, and 30% of the varieties had stalks of 95–110 cm.

We found that almost all KSR varieties had strong resistance to pests and diseases and had good quality. In addition, almost all KSR varieties were used in the same way to cook steamed sticky rice, brew glutinous rice wine, and make glutinous rice cake, salted fish, and meat [40]. However, in our investigation of local farmers, we found that certain varieties have particularly desirable characteristics. For Baixianghe, which had the largest planting area in Kengdong village, the most prominent feature was the strong aromatic flavor. Huangshanxue could be cooked with red jujube or rice bean together and was thought to have medical value by local people. Shuiniumao was a unique variety suitable for planting in the cold and in the water contaminated by iron at high and low altitudes, and it had red grains. Because of these characteristics, the Dong people liked to use Shuiniumao for steamed red rice on salted fish or meat. Zhaoxingheinuo was not only suitable for cold paddy fields, but was also used to make black rice cakes, eliminating the steps of dyeing black glutinous rice for the Black Rice Festival (Chinese calendar April 8th). Goucengao was very stable in yield, had very strong resistance to cold, and had been cultivated in Biapa, Jianghua, and Xinsheng villages from Congjiang County for hundreds of years. Gouyue was also very stable in yield and quality and had strong resistance to disease, causing it to have the largest planting area and the longest history of cultivation in Longjiang village in Congjiang County.

### Investigation of KSR in typical ethnic villages

**Fig. 3:**
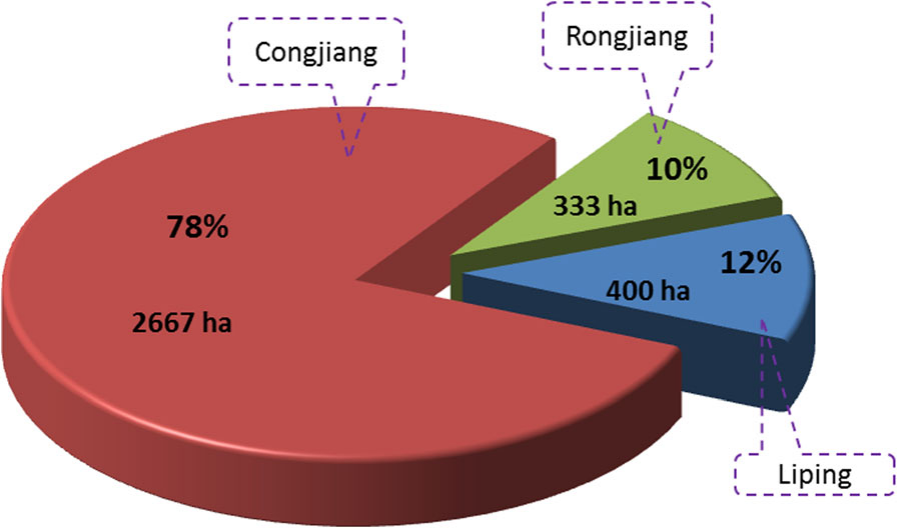
Planting area and proportion of KSR in 3 counties

In 2015, the total area of KSR in the main areas of Qiandongnan—Liping, Congjiang, and Rongjiang counties—was about 3400 ha. Congjiang had 2667 ha, accounting for 22.5% of the total paddy fields in that county (10,000 ha); Liping had 400 ha, accounting for 2% of the total paddy fields in that county (20,000 ha); and Rongjiang had 333 ha, accounting for 3.2% of the total paddy fields in that county (10,533 ha). The cultivation area of KSR in Congjiang was the highest, 6.7 times that of Liping and eight times that of Rongjiang (Fig. 3).

We selected 33 typical villages with different ethnic groups, elevations, and population sizes in 15 towns from Liping, Congjiang, and Rongjiang counties of Qiandongnan Prefecture, including 25 Dong ethnic villages and 8 Miao, Shui, Yao, and Zhuang ethnic villages; information about each village is shown in Table 3. The elevation ranged from 240 to 935 m, population size ranged from 509 to 4175, and per capita paddy field area ranged from 0.01 to 0.07 ha. The results showed that KSR cultivation area was the largest in Longjiang, Jianhua, Zhanli, and Dingdong villages in Congjiang County; the ratio of KSR to total paddy area was above 80%. The ratio of KSR to total paddy area ranged from 50 to 70% in 6 villages, 4 of which (Zaizhuan, Biapa, Xinsheng and Zaigong) were in Congjiang County. The cultivation area of KSR in Kengdong and Huanggang was larger than other villages of Liping County, accounting for 50% and 63% of the total paddy field area of Liping County, respectively. The area ratio of KSR in ten villages ranged from 20 to 40%; these villages were Shuangjiang, Deshun, Shangzhong, and Leidong in Liping County and Gaozeng, Cuili, Bingmei, and Jiabang in Congjiang County. There were 12 villages with cultivation areas under 20%, and 5 villages in Rongjiang County accounted for only 3–4.5% of the total paddy field area.

**Table 3:** Information of villages investigated

County	Town	Village	Ethnic group	Elevation (m)	Population	Per capita paddy area/ha	KSR area ratio (%)	Number of accessions	Total KSR area/ha
Liping	Shuangjiang	Kengdong	Dong	380	1580	0.07	50	10	400 2%
		Huanggang	Dong	750,735	1600	0.07	63	21	
		Miedong	Dong	448	1400	0.07	25	3	
	Yandong	Yandong	Dong	549	4175	0.06	7.0	3	
		Zaigong	Dong	330	1104	0.02	50	2	
		Xiaozhai	Dong	366	594	0.03	23	3	
	Zhaoxing	Zhaoxing	Dong	458	1770	0.04	15	6	
	Deshun	Pingfu	Dong	520	952	0.07	10	1	
		Deshun	Dong		1744	0.05	29	2	
	Shangzhong	Jiaodong	Dong&Miao	922	537	0.04	20	3	
	Leidong	Leidong	Yao&Shui	290	753	0.04	31	2	
		Cengong	Dong	570	732	0.05	29	2	
Congjiang	Gaozeng	Jianhua	Dong	800	777	0.05	83	10	2667 22.5%
		Zhanli	Dong	380	829	0.07	80	29	
		Biapa	Dong	600	1200	0.04	65	10	
		Xinsheng	Dong	582	1259	0.03	60	7	
		Xiaohuang	Dong	618	3700	0.04	33	6	
	Cuili	Baiweng	Yao	544	809	0.01	40	5	
		Zaizhuan	Dong	500	1070	0.05	70	5	
		Gaowen	Zhuang	470	607	0.04	32	4	
	Bingmei	Biasha	Miao	550	2336	0.03	40	3	
	Yongli	Longjiang	Dong	430	2190	0.05	86	6	
	Xishan	Dingdong	Dong	240	2000	0.05	80	5	
	Tingdong	Changzhai	Miao		1093	0.02	11		
		Jiali	Miao		2154	0.02	11		
		Jiadi	Miao		509	0.03	11		
	Gangbian	Yinping	Zhuang&Miao	277	1159	0.02	15		
	Jiabang	Dangniu	Dong		913	0.03	30		
Rongjiang	Zaima	Zaima1	Dong	576	2710	0.03	3.4	1	333 3.2%
		Zaima2	Dong	576	1894	0.03	4.0		
		Gaodong	Dong	778	2481	0.05	4.5		
		Xiaoli	Dong	935	1022	0.03	3.2		
		Bakuang	Dong	432	647	0.02	3.0		

**Fig. 4:**
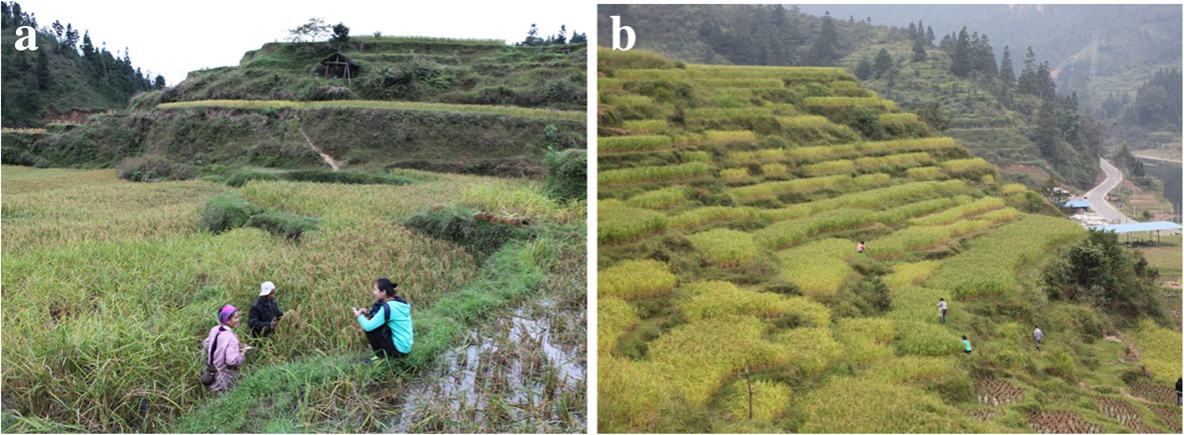
Paddy fields of KSR in **a** Huanggang village and **b** Yinping village

The cultivation area of KSR in Dong villages was larger than in villages inhabited by members of other ethnic groups. The cultivation area in 6 villages—Longjiang (87.9 ha), Dingdong (85.3 ha), Huanggang (66.7 ha), Kengdong (52.7 ha), Xiaohuang (48.9 ha), and Zhanli (48.7 ha)—was more than 48 ha, reflecting high demand of the Dong people for KSR. Additionally, villages that had a higher planting area of KSR had more KSR accessions; generally speaking, these villages had 6–7 accessions, and some villages retained up to 20–30 KSR accessions. Paddy fields of KSR in Huanggang and Yinping villages are shown in Fig. 4.

### Farmers’ attitudes concerning the development and protection of KSR

We randomly selected 56 farmers from Sizhai (26 farmers) and Huanggang village (30 farmers) in Liping County for the questionnaire. The distributions of gender, age, and cultural level of respondents are shown in Table 4. 51.8% of farmers were male; 48.2% were female. Among the respondents, the age range of 36 to 60 years accounted for the largest percentage at 48.2%, over 60 accounted for 26.8%, and under 36 accounted for 25% of the total respondents. The majority of respondents had a low education level: 73.2% of the total respondents were illiterate and had only finished primary education, the percentage of junior high school was 21.4%, and only 5.4% had completed senior high school. The results showed that the gender, age, and educational attainment were distributed reasonably, which could reflect the overall attitude of local villagers to the survey.

**Table 4:** Analysis of social characteristics of investigated respondents

Social characteristics	Classification	Quantity	Percentage
Gender	Male	29	51.8
	Female	27	48.2
Age group	≤ 20	2	3.6
	21–35	12	21.4
	36–60	27	48.2
	≥ 61	15	26.8
Education level	Illiteracy	26	46.4
	Primary School	15	26.8
	Junior High School	12	21.4
	Senior High School	3	5.4

**Fig. 5:**
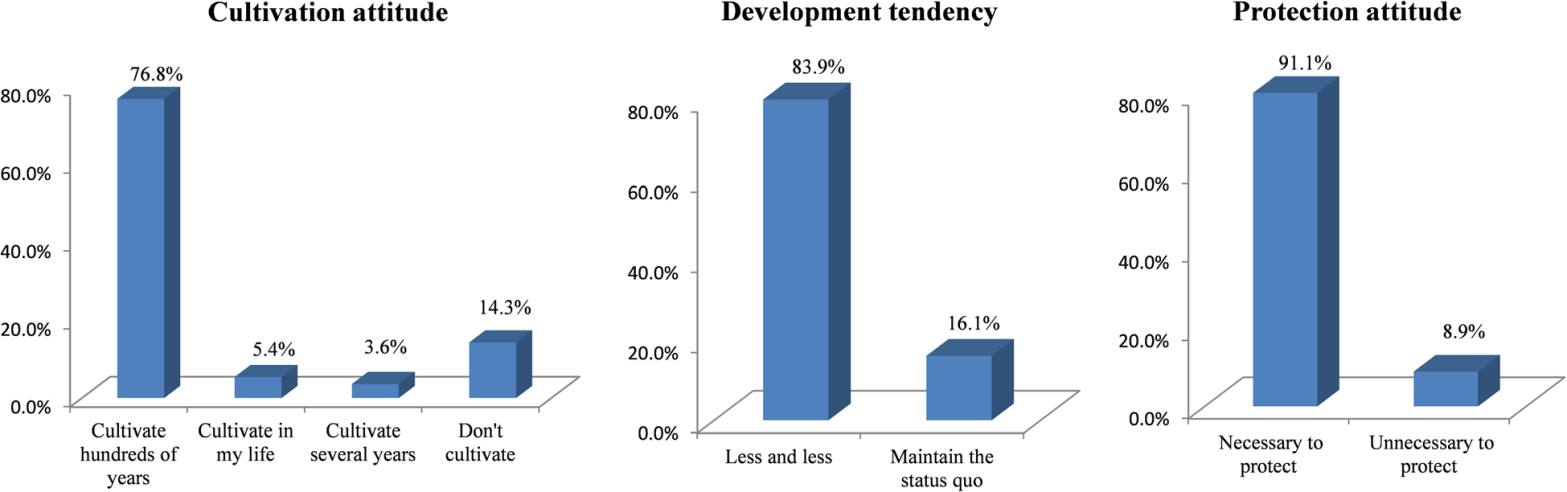
Dong farmers’ attitudes to the cultivation, development tendency and protection of KSR

Dong farmers’ attitude to the cultivation, development tendency, and protection of KSR is shown in Fig. 5. For *cultivation attitude*, 85.7% of respondents said they will continue to cultivate KSR, of which 76.8% said they will the future generations to continue cultivation and to protect this traditional rice landrace. 14.3% of respondents will not continue to cultivate. For *the reason of continuing to cultivate KSR*, all respondents (100%) believed that ethnic traditional culture and social customs were the main factors for continuing to cultivate KSR, including using KSR for daily food, festival celebrations, belief sacrifice, gifts, and the “Rice-Fish-Duck Symbiotic System”. A small number of farmers (4.2%) also believed that suitability to the natural environment and resistance to diseases and pests were reasons to continue to cultivate KSR. For *the reason of stopping to cultivate KSR*, opinions mainly focused on economic, management and policy reasons, including low yield, time and labor consumption, and the government policy of promoting hybrid rice and other glutinous rice. In addition, 12.5% of respondents thought the social customs change and unsuitability to the local environment also led to the decrease of KSR cultivation. For *development tendency*, most farmers (83.9%) thought that KSR varieties will decrease and will be abandoned, while 16.1% thought that the status quo will be maintained. Since the Chinese government policy of reforming and opening up from 1980s, people in the surveyed villages had been greatly influenced by foreign culture, including the acceptance of hybrid rice varieties. Most Dong people were gradually accepting foreign culture and hybrid rice and other modern crop varieties, so the majority people hold a negative attitude toward cultivation of traditional KSR varieties. For *protection attitude*, almost all farmers (91.1%) thought that it is necessary to protect rice landraces, but other farmers thought it is unnecessary to protect KSR, because hybrid glutinous rice could also meet their needs.

Based on the attitudes of local farmers with different social characteristics to the cultivation, reasons for conservation, reasons for abandonment, development tendency, and protection of KSR, we analyzed the correlation between different social characteristics and attitudes of farmers. The correlation coefficient and significant level are shown in Table 5.

**Table 5:** Correlation analysis between farmers with different social characteristic and their attitudes on KSR

Questions	Gender	Age	Education level
Cultivation attitude	− 0.121(*p* = 0.372)	0.123(*p* = 0.366)	0.021(*p* = 0.876)
The reason of continuing to cultivate KSR	0.000(*p* = 1.000)	− 0.110(*p* = 0.455)	0.027(*p* = 0.853)
The reason of stopping to cultivate KSR	0.020(*p* = 0.963)	− 0.323(*p* = 0.434)	0.434(*p* = 0.283)
Development tendency	− 0.228(*p* = 0.092)	− 0.137(*p* = 0.314)	0.015(*p* = 0.913)
Protection attitude	− 0.051(*p* = 0.706)	0.166(*p* = 0.223)	− 0.086(*p* = 0.528)

**Fig. 6:**
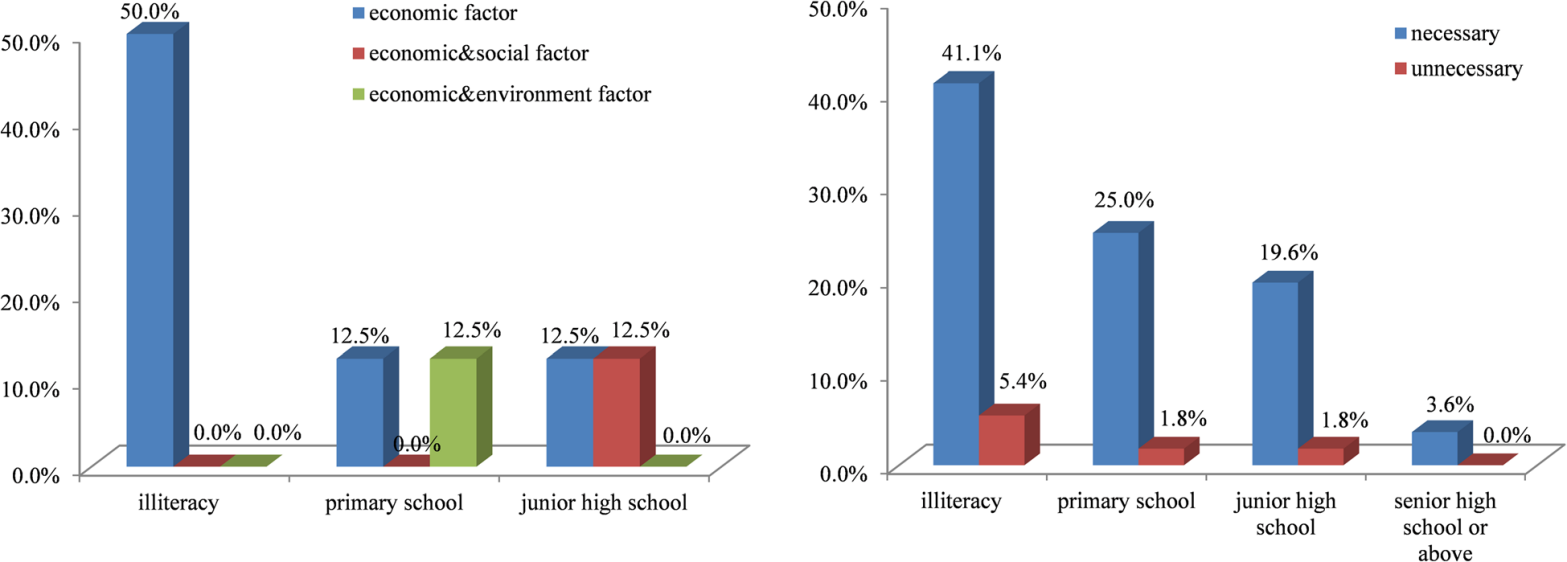
The attitude of different education level to the abandonment and protection of KSR

**Fig. 7:**
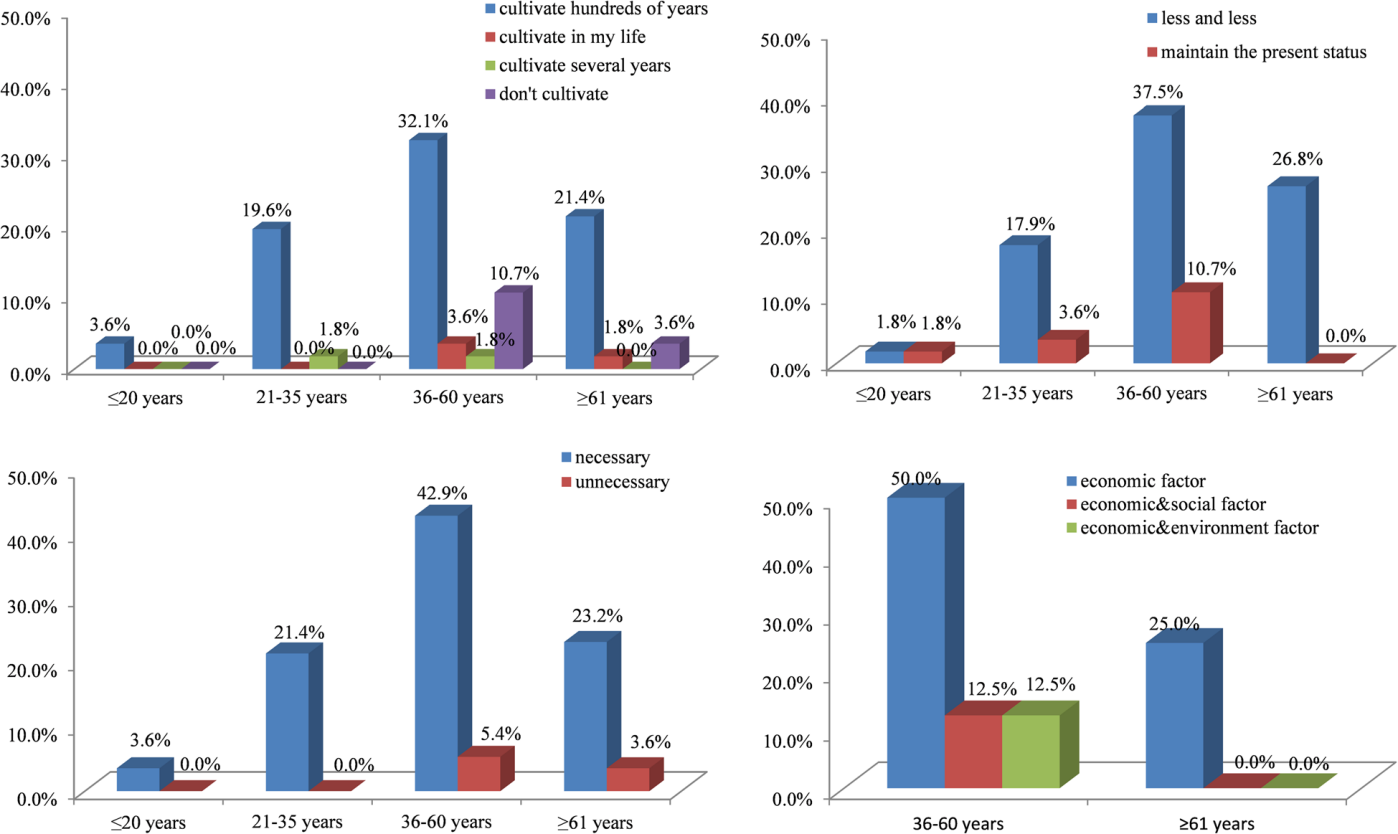
The attitude of different age group to the cultivation, development, protection and abandonment of KSR

**Fig. 8:**
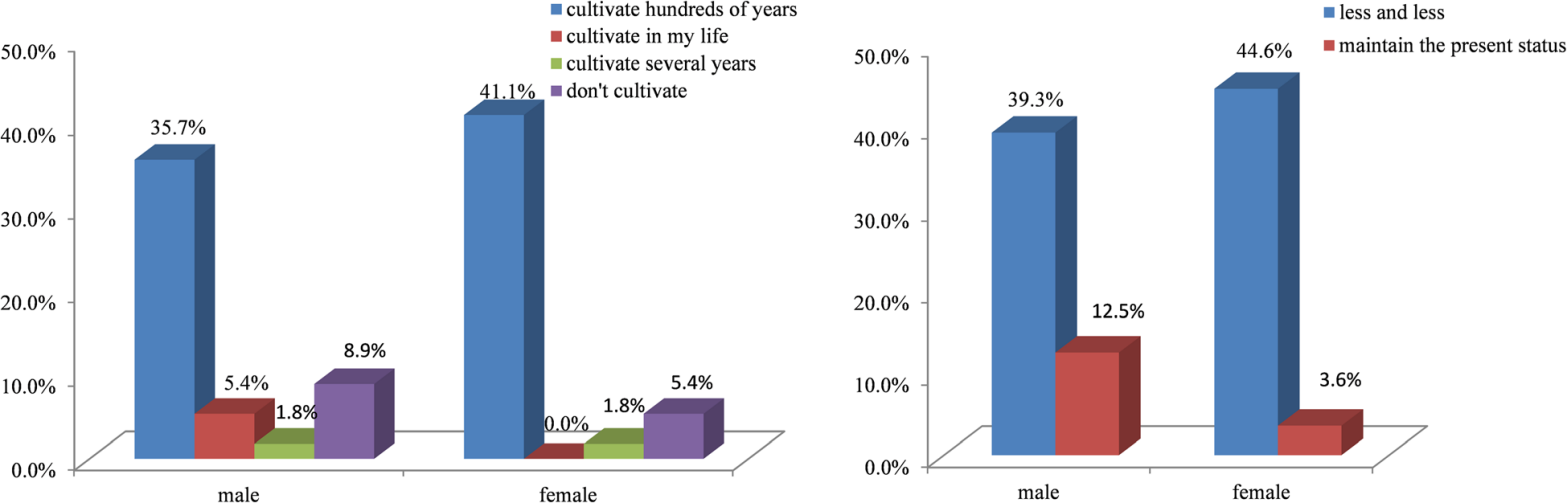
The attitude of different gender to the cultivation and development of KSR

We found that the characteristics of gender, age, and education level were correlated with farmers’ attitude to KSR cultivation and protection; however, there were no significant differences between two aspects. Though farmers were female and male, young, middle-aged and elderly, and had different levels of education, all of them hold a similar attitude: most of them will continue to cultivate KSR, and they hold the view that KSR varieties will decrease gradually and need to be protected. But some farmers still hold different views. In particular, the correlation coefficient between education level and the reason of stopping to cultivate KSR was the highest at 0.434. This result showed that all of primary and junior high school level farmers think the reason of stopping to cultivate KSR is mainly due to social and environmental factors in addition to economic factors (including taste habits, the function of KSR as a gift has changed, KSR is unsuitable to local climate environment), while illiterate farmers only considered the importance of economic factors. 9.3% of under junior high school thought it was unnecessary to protect KSR, while all senior high school thought it was necessary (Fig. 6). Different age groups of farmers also had close correlations with the cultivation (0.123), abandonment reason (− 0.323) and development tendency (− 0.137), and protection attitude (0.166). The young people were more positive to cultivating KSR than middle-aged and elderly people. In the 36–60 age range, the number of farmers cultivating KSR was three times higher than farmers not cultivating KSR, and above 61 years, the number of farmers cultivating KSR was six times higher than farmers not cultivating. 25% of 36–60-year-old farmers thought the reasons for abandoning KSR were affected by social and environmental factors in addition to economic factors, while all of farmers older than 60 years thought it is only due to the economic factors. All farmers older than 60 thought the development tendency of KSR will be decreasing, while others thought KSR will be decreasing or maintain the present status. Farmers under 35 thought it is necessary to protect KSR, while 11.9% of farmers above 36 thought it is unnecessary to protect KSR (Fig. 7). For the attitude of cultivation and development tendency, gender had a correlation coefficient of − 0.121 and − 0.228, respectively. More female farmers (41.4%) will cultivate a longer time than male farmers (35.7%), and fewer female farmers (5.4%) will not cultivate KSR; 77.8% of male farmers thought KSR will maintain the present status, while 22.2% female farmers hold the same view; thus, the male farmers were more optimistic than the female farmers (Fig. 8).

In conclusion, the young (under 35) and people with a higher level of education thought it is more necessary to protect KSR varieties. The middle-aged (36–60) and people with a higher level of education thought that social and environmental factors also affect the abandonment of KSR. Male and young farmers were more optimistic to the development tendency, having the idea that KSR will maintain the present status, while the female and old farmers were more pessimistic about the development tendency of KSR. Compared to male farmers, female farmers will cultivate KSR for a long time and fewer will abandon KSR genetic resources. The young and elderly were more positive toward cultivating KSR. Therefore, a high level of education and female, young, and old farmers will play more important roles in the cultivation and protection of KSR.

## Discussion

### Factors influencing the conservation of KSR

The conservation and abandonment of KSR genetic resources were influenced by many factors. Based on the results of the questionnaires in Sizhai and Huanggang, we found that ethnic traditional culture, social customs, economic benefits, environmental conditions, management, and policy are the factors influencing the cultivation and protection of KSR. Of these, ethnic traditional culture and social customs are the main factors influencing conservation of KSR, while economic, management, and policy are the main factors influencing abandonment of KSR. However, it was unclear how these factors affect the conservation and abandonment of KSR. Therefore, we discussed these different influencing factors in detail.

#### Ethnic traditional culture and social customs

##### Food culture and taste habits

**Fig. 9:**
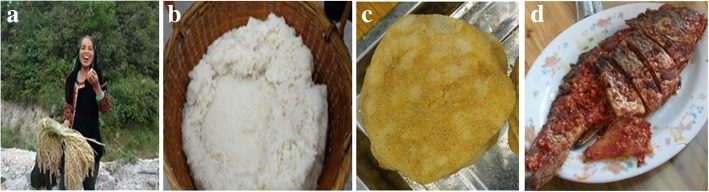
KSR rice in Dong’s daily food culture: **a** rice ball for lunch, **b** steamed rice, **c** glutinous rice cake, and **d** pickled fish with steamed rice

The Dong people strongly preferred glutinous rice, and many foods in their daily life cannot be made without KSR [21]. In our findings, the most important food was steamed KSR, with many outstanding characteristics including softness, even with cold rice; stickiness; strong fragrance; and a good nutritional profile. Currently, Dong people from Huanggang and Kengdong villages still eat KSR every day; they were accustomed to the taste, and this kind of sticky rice was convenient to carry, because the villagers often knead it into a sticky rice ball for lunch. Dong ethnic groups from Zhaoxing, Deshun, Leidong, Yandong, and Shangzhong villages were fond of making oil-tea (oil, fried KSR, and tea) for breakfast and afternoon tea. People from Kengdong, Yandong, Xiaohuang, and other Dong villages liked flat rice, which was made of fresh rice when the grain is a little green, through frying or steaming. In addition, Dong people also liked pickled fish, meat, and vegetables using steamed KSR, and boiled fish and vegetables and pickled salted duck eggs with the fermentation water from washing KSR, because the taste was better than hybrid rice. The pictures of KSR in Dong’s daily food culture were shown in Fig. 9.

##### Festival celebration

**Fig. 10:**
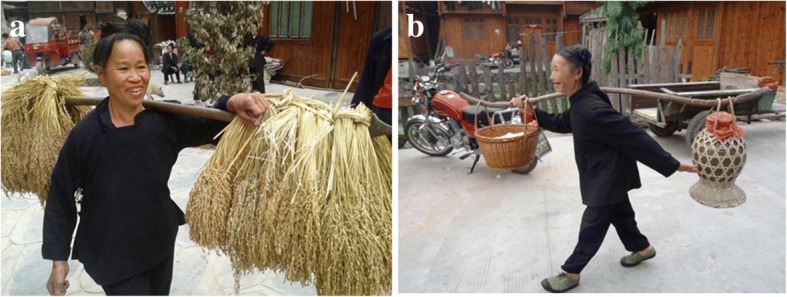
**a** Ten bundles of KSR spike and **b** A jar of KSR wine and a basket of steamed KSR rice to celebrate a baby that is 1 month old

We found that KSR has become an essential good for Dong people celebrating festivals and visiting relatives and friends. After young men and women were married, the groom’s family must prepare a large number of rice cakes, dumplings, or steamed rice as a gift to send to the bride. Rice cakes were made of hundreds of pounds of KSR, and the bride’s family then sent this gift to other villagers to announce the engagement. During Dong’s festivals from the lunar calendar, including March 3, May 5 (Dragon Boat Festival), June 6, September 9, and the Spring Festival, local people used KSR as raw materials to make different rice foods. For example, in the Black Rice Festival (Chinese calendar April 8th), Dong people in Zhaoxing and Yandong villages dyed KSR black with black leaves and then steamed it. Farmers who ate black rice pretend to eat cow dung, in order to appreciate their hard work and show their respect to the cow. Dong people in Pingfu village made Dongguo (a traditional food made of KSR) to entertain guests at the lunar celebration on October 12. The “Wrestling Festival” in Sizhai village, “Shout Day Festival” in Huanggang, and other Dong festivals also used KSR. Dong people considered KSR as the most precious rice, and thus, they sent KSR for the red and white wedding gifts. For the “full moon wine” celebration (to celebrate a baby that is 1 month old) in Zhaoxing and Sizhai village, Dong people sent KSR as gifts (Fig. 10), and when the guests left, the host will pack some steamed KSR.

##### Belief culture

In the Dong villages we investigated, some Dong people only cultivated very few KSR varieties by themselves; they did not buy or exchange it, due to the important position of KSR in Dong’s belief culture. In some of the Dong people’s sacrifices and rituals, KSR was necessary, and it could not be substituted by other glutinous rice or hybrid rice because sacrificial ceremonies must use local and traditional food; otherwise, they will not feel devout. For instance, the whole process of offering sacrifices to tree gods must use KSR and its wine as tributes. When elders died, the most important food to worship the dead was KSR and sour fish. Relatives and friends mourned the dead or attended the funeral ceremony, taking a spike of KSR in the hands, to show that wherever they go, whether living or death, everyone could eat KSR. The culture of faith is important for continuing to cultivate KSR.

##### Traditional farming system

**Fig. 11:**
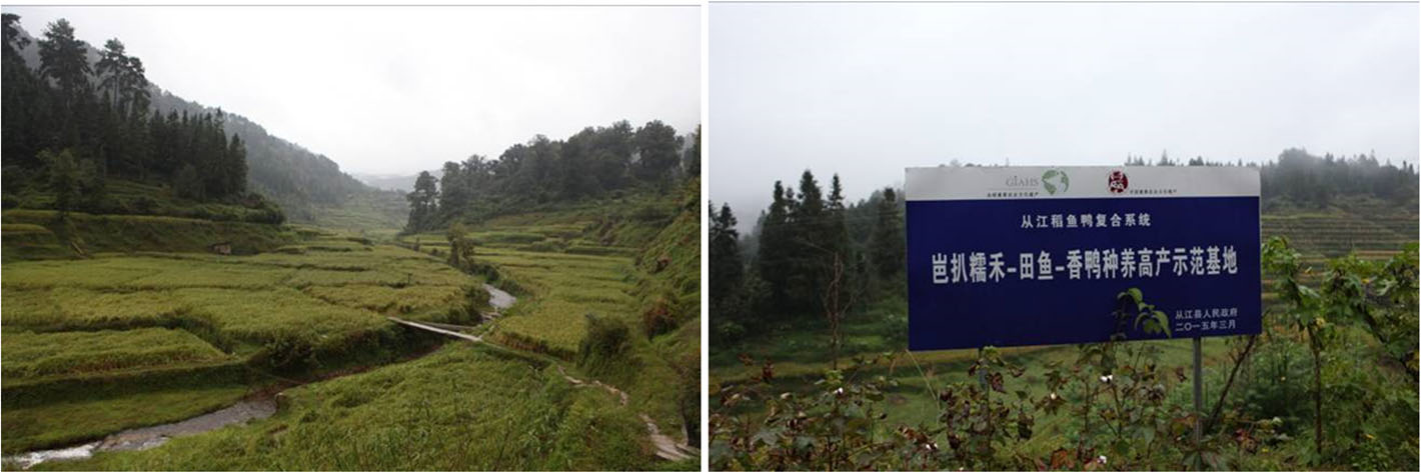
Rice-Fish-Duck Symbiotic System in Biapa village of Congjiang County

The traditional farming system of Dong ethnic groups also influenced the cultivation and protection of KSR genetic resources. The Dong people’s “Rice-Fish-Duck Symbiotic System” had a long history of 1400 years and was the typical traditional eco-farming method used by the Dong people (Fig. 11). Around Grain Rain (6th solar term) in spring, the rice seedlings were transplanted to the paddy fields and fish fry were released into the fields at the same time. When the fry grew to 10 cm, ducklings were released into the paddies. The rice paddies could provide fry and duckling with a hydrophytic habitat with rich food sources including weeds, insect pests, and plankton, and in turn the duck mature provided nutrition to the paddy, reducing the use of pesticides and chemical fertilizer. This eco-system had multiple benefits for the economy, ecology, society, and culture. In practice for hundreds of years, the “Rice-Fish-Duck Symbiotic System” allowed for a harmony between people and nature based on traditional knowledge [41]. As it was a sustainable production model, this “Rice-Fish-Duck Symbiotic System” was selected as a case of Globally Important Agricultural Heritage under UN/FAO. This system enhanced the yield and quality of KSR, promoted the enthusiasm of farmers to cultivate KSR, and protected the valuable crop varieties of Dong people.

#### Environmental factors

KSR adapted to environments with thick fog, high humidity, and short periods of sunshine and was generally more resistant to stress than other rice varieties, which was the main reason suitable to be planted in the natural environment of Qiandongnan [7]. Most KSR varieties had awns, which can resist the harm of birds and animals, and were rarely infected by plant pathogens [12]. Most KSR varieties adapted to the soil under medium or low levels of fertilization, which can guarantee production without the use of chemical fertilizers or with just a small amount of manure. Some KSR varieties, including Guiyanghe, Rongzhuhe, and Shuiniumao, can also be planted in the fields that are deep, cold, and contaminated with iron. Most KSR varieties had a strong ability to resist pests and grew well without chemical pesticides. In our field research, we found that some varieties, including Huangshanxue, Shuiniumao, and Gaoyanghonghe, were not affected by diseases or insect pests because of the strong disease-resistant ability. Some varieties can be robust and grow normally even in low light, such as Huanggangyangnong. Some He varieties were highly drought tolerant without reductions in yield. Specifically, the variety of 120-days He, 130-days He from Jianghua village in Congjiang County, and 60-days and 70-days He from Huanggang in Liping County had a shorter growth duration and thus were suitable for planting in paddy fields undergoing drought.

#### Economic factors

Many economic factors influenced the cultivation of KSR. First, KSR had a relatively higher market price, 2–2.5 USD/kg, while the price of hybrid rice was only 1–1.5 USD/kg. Second, the rice stalks of KSR were also harder than hybrid rice, allowing the Dong people to not only make clean and durable brooms, but to also make rice dumplings and rice cakes. Rice straw ash can also be used as fertilizer or dyestuff to provide raw materials for daily life. The rice straw can even be sold for 0.6 USD/kg in some areas. Third, KSR can be used to make many by-products. The “Rice-Fish-Duck Symbiotic System” not only grew KSR, but also produced fish and duck meat as by-products. Moreover, the upper layer of “sour soup” after fermentation of KSR was used to cook fish and vegetables, and the lower sediment was used to wash hair, which was a cost-saving measure.

### Factors influencing the abandonment of KSR

#### Economic, management, and policy factors

##### Economic benefits

Under the same management conditions, the yield of KSR was about 4500–5250 kg/ha. *Indica* rice could increase yield by 20–30%, and hybrid rice could increase yield by 70–80%. Farmers cultivating *indica* or hybrid rice cannot only satisfy daily consumption, but can also increase income by selling excess rice. Currently, the farmers cultivating KSR were only self-sufficient and got low incomes.

##### Social management

**Fig. 12:**
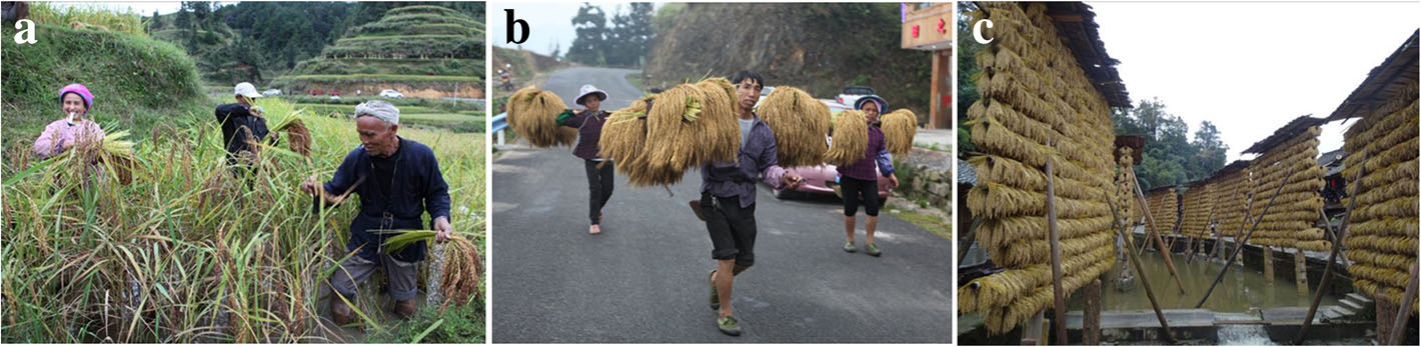
**a** Zhai He in Huanggang village. **b** Tiao He in Dangniu village. **c** Heliang in Zhanli village

Overall, processing KSR was more complicated than processing *indica* or hybrid rice. KSR must be harvested manually, which was time-consuming and laborious, while hybrid rice can be mechanically harvested, saving time and effort. KSR harvesting required a special tool, “half-moon pliers”, and must be reaped by hand (Zhai He). After the harvest, rice ears were knit together with rice straw, then picked up and carried on the shoulders (Tiao He), hung on a bamboo shelf to dry in the sun (He Liang), and finally stored in a granary (Fig. 12). These methods were similar to what is done with Tibetan dry grass.

##### Government policy

Compared to KSR (*japonica* rice), *indica* rice had a higher yield; therefore, the Liping County government had switched from cultivating KSR to cultivating *indica* rice since the establishment of the People’s Republic of China. This change caused the cultivation area and number of varieties of KSR to gradually decrease. In 1950s, the KSR cultivation area in Liping County was larger, accounting for about 75% of the total rice area; in these areas, KSR was a primary food for the Dong people. After the 1980s, the promotion of hybrid rice cultivation caused the KSR planting area in Liping County to decline sharply, and by 2013, the total area of KSR was only about 2% [21].

#### Environmental factors

Environment factors mainly reflected in that some biological characteristics of KSR made it unsuitable to local environments, such as a long growth period, high water demand, long stems (KSR is not resistant to lodging), and reduction in output in dry years. However, hybrid rice possessed a short growth period, lodging resistance due to short stems, and was typically more suitable in local natural environments. In addition, the maturity period of *indica* rice or hybrid rice was 1 month earlier than that of KSR. Because of this, the soil can also be used to grow vegetables or fertilizer after harvesting *indica* rice or hybrid rice, so as to expand the replanting area and improve the land use rate. Some KSR varieties were gradually being phased out because they were unsuitable for the local climatic conditions. For example, in Huanggang village, the Old-Liezhuhe variety was replaced by New-Liezhuhe because Old-Liezhuhe is not suitable for growing in well-lit paddy fields. In addition, the Niumaohe and Jindongnuo varieties were also gradually being abandoned by local farmers because they were easily affected by climate conditions, had a very long stem (> 160 cm) and thus no resistance to lodging, leading to low yield.

#### Ethnic traditional culture and social customs

KSR had been a staple food for the Dong people for thousands of years and had been integrated into various aspects of their life, and people formed unique traditional culture and social customs around KSR. However, due to the reform and opening-up policy of Chinese government in the 1980s, combined with increases in convenient travel and communication and the influence of foreign culture, the importance of KSR in the daily life of the Dong people has declined. In the past, farmers harvested KSR, not only to get the grain, but also to harvest pliable and strong rice straw to make many byproducts. Due to changes in culture and social customs, the diet, clothing, and tools of the Dong people had gradually been replaced with the products of modern civilization. For example, the traditional staple food of the Dong ethnic group is glutinous rice, mainly KSR, but they currently eat non-glutinous rice. Previously, the Dong people used straw sandals woven with KSR straw, but these are no longer used now. Also, the Dong people used to wash their hair and clothes with the ash burned with rice straw, and now they use shampoo and powder or liquid detergents. KSR straw was previously used as packaging for gifts or to make glutinous rice cake when visiting friends and relatives, but now young people prefer to use plastic or paper bags as packaging materials. In addition, the changes in measuring tools and units have also affected the cultivation of KSR. The previous measuring tools of Dong people were denx (1 denx = 10 bundles of KSR), and they also measured land with denx. However, since the Chinese government used acres as a unit, young people who had received education also use acres. Compared to the past, KSR could be replaced by many modern products, which was an important internal reason for decreases in the cultivation area and number of varieties of KSR.

### The influence of Dong traditional culture and social customs on the formation of KSR characteristics

KSR has many unique characteristics including strong glutinous trait, difficult to thresh, strong resistance to adverse conditions as well as to diseases and insect pests, and moderate yield [12]. The formation of these characteristics were influenced by Dong ethnic culture and social customs for thousands of years.

#### Strong glutinous trait

Most farmland of the Dong people was located on hills and far from villages. Glutinous rice was useful because Dong farmers could use it to make rice balls that are easy to carry with them for lunch. KSR varieties also had a long shelf life, and cold rice did not harden, making it convenient to carry. Moreover, strong glutinous rice was a good raw material for making foods including glutinous rice cakes, and rice wine used in festivals of the Dong people. Festival food was one important reason to preserve the strong glutinous trait of KSR.

#### Difficult to thresh

At harvesting time, a specialized tool, the “half-moon plier”, was necessary to artificially harvest KSR. Following harvest, KSR was tied into many bundles of panicles, dried on homemade shelves, and stored in granaries. These practices were necessary because the cold and humid climate in Qiandongnan will cause the grain to mildew, and it will not be edible. While the method of hanging to dry could mitigate this problem, it also increased shelf life and retained the fragrance and freshness of the rice. Dong people also liked to send a few bundles of KSR as gifts for different festivals. Furthermore, the rice straw was very long and hard and was not only suitable for processing straw rope, weaving straw shoes, making Zongzi, but could also be burned into grass ash that was a raw material for dyeing cloth.

#### Strong stress resistance

Most KSR varieties can grow normally in harsh environmental conditions, such as cold climate, soil contaminated with iron, or saline-alkali paddy fields, without negative effects on production or quality. These varieties were suitable to local adverse environment and were preserved by local farmers. At the same time, these traits resulted in some varieties not being tolerant to fertilizer, and with more fertilizer hindering growth.

#### Strong resistance to diseases and insect pests

KSR had a strong ability to resist diseases and pests, including rice blast, brown planthopper (BPH), and bacterial leaf blight. The Dong people had retained the resistant varieties in the process of breeding, and abandoned the varieties without resistance. Some varieties had long awns, allowing them to effectively resist birds and animals and rarely be infected with diseases and pests [12].

#### Moderate yield

KSR had an average yield of 4500–5250 kg/ha, with a maximum of 6000 kg/ha. While its yield was far lower than hybrid rice varieties (130,000 kg/ha), the yield was not much lower than the general breeding varieties (8000 kg/ha). The milled rice rate of KSR was 10% higher than hybrid rice. This production could meet daily consumption requirements for the Dong people and was enough for other traditional customs. Therefore, the moderate yield characteristic of KSR was an important reason for Dong villagers to maintain KSR cultivation.

The natural climatic conditions in Qiandongnan region, along with traditional culture and social customs, were the main influencing factors of KSR’s unique characteristics. For example, in Huanggang village of Liping County, the distance from home to paddy field was around 1–2 h and Dong people had been cultivating and eating KSR since villages were first established (about 800 years ago), not only to meet their physical demand, but also because this glutinous rice was well-suited to the local climate. Even with climate change, the planting area of KSR had remained around 65% of total paddy field area, and the yield had remained relatively stable. Moreover, 25 varieties were reserved to plant at different elevations and conditions in Huanggang. Dong people in Huanggang village had taken their farming wisdom and engaged in a life style of cultivating and eating only KSR, because it could meet the demand of production and life, and it allowed them to breed excellent KSR varieties [42].

### The relationship between ethnic traditional culture, social customs, and conservation of KSR

As an important genetic resource, traditional landraces were the foundation of agricultural biodiversity and played an important role in the generation and development of ethnic traditional culture and knowledge. Ethnic traditional culture and knowledge also promoted the protection and sustainable utilization of genetic resources, while maintaining and enriching the diversity of genetic resources. Therefore, the preservation of genetic resources and the protection of ethnic traditional culture were interdependent and mutually reinforcing relationships. Relevant studies showed that cultural backgrounds were closely related to the diversity of crop varieties, and understanding the cultural background was an important premise for protecting the diversity of crop varieties [43, 44]. For example, the preservation of rice landrace diversity in Yunnan, China was closely related to the highly heterogeneous ecological environment and ethnic cultural customs where rice was grown [45, 46]. Rice farmers in Asia had grown thousands of rice varieties with different flavors, medicinal, and cultural values to meet the needs of different food cultures [47–49]. In addition, some scholars in Nepal, the Philippines, Vietnam, and other Southeast Asian countries also suggested that ethnicity and rice culture had a close relationship with the diversity of rice landraces [44, 50]. Other researchers also found that a southeast Asian origin for glutinous rice was consistent with Asian cultural practices [51]. An important culinary and cultural component throughout east Asia, glutinous rice was generally reserved for use in festival foods and desserts, although it also served as the staple food in upland regions of Southeast Asia [52, 53]. Lei et al. [54] also found the prototypical ethnic cultures had a positive impact on the conservation and utilization of glutinous rice diversity in Qiandongnan of Guizhou Province.

As one of the most distinctive rice landraces in Guizhou Province of China, KSR had a positive effect on Dong’s food culture and traditional customs. Dong people were inseparable from KSR: KSR was used in festival celebrations, ritual activities, weddings, funerals, and other cultural customs, which promoted KSR planting and use by Dong people for thousands of years. KSR was not only a simple food crop, but is also the essence of Dong culture. In a certain sense, Dong culture was KSR culture. The continuous planting of KSR had protected the cultural customs of the Dong people. Some villages were still cultivating KSR up to 80% of paddy area, because the local natural environment conditions were suitable, and more importantly, KSR was a part of Dong traditional culture. These villages had maintained the cultivation of KSR for thousands of years.

In 2007, the Dongxiang Rice Limited Company in Liping County developed organic KSR under the support of government, and established the Kengdong KSR cooperatives. These cooperatives took the development mode of “Company-Cooperatives-Farmer” and formed a standardized system of cultivation, management, purchase, and market sale of KSR, which increased the farmers’ income, also effectively protected KSR resources, and inherited traditional culture and social customs of the Dong ethnic group. In 2009, “Liping KSR” won National Protection of Geographical Indications. In 2011, Liping County government drafted “Protection and Management Measures of Liping KSR”, to protect KSR in the form of laws and regulations, and to guarantee the Liping brand and quality characteristics of KSR. KSR was the Dong’s most distinctive food crop, and it was currently sold throughout China as a local characteristic product, causing more and more people to realize the food and culture value of KSR.

### Protection measures for KSR

The protection of crop variety resources included two methods, in situ on-farm conservation and ex situ gene-bank conservation. Previous studies of crop landraces under on-farm and ex situ protection had summarized some similar conclusions using molecular markers and morphological analysis. Different researchers had taken the materials of rice from China [55, 56], Vietnam [57], and Guinea [58]; corn from the USA [59] and Mexico [60]; kidney beans (*Phaseolus vulgaris* L.) from Italy [61]; common bean from Nicaragua [62]; barley from Syria [63]; and sorghum from Niger [64], and studied the genetic and morphological diversity within populations of local varieties preserved in a gene-bank and farmlands. They found that on-farm conservation maintained or enriched genetic heterogeneity and diversity. Based on SSR markers for four pairs of KSR varieties, four varieties from 1980 were preserved ex-situ in gene-banks, and the other four varieties from 2014 were collected from on-farm conservation in Guizhou Province. The results showed that, compared with gene-banks, on-farm conservation can effectively promote allelic variation of traditional rice landraces, increasing the genetic heterogeneity and diversity [30]. On-farm conservation not only allowed for continued evolution of the crops in the original habitat, but also included the participation of farmers in selection. These practices promoted ethnic culture and social customs, allowed for the preservation and use of crop variety resources, and effectively increased the genetic background and genetic diversity.

At present, the collection and protection of rice germplasm resources in Guizhou is a combination of national and local gene-banks. However, due to the weak economic and technological base, Guizhou Province had not established stable research funds for the preservation and use of crop genetic resources [65]. Related studies [66, 67] found that most rice landrace resources were distributed in remote and poverty-stricken areas with rich biodiversity. In recent years, in order to protect the ethnic traditional culture and agricultural biological resources, the Chinese government established a committee for ad hoc basic study of major national science and technology projects. These groups carried out systematic investigation and collection of crop landraces in 41 counties (cities) and 10 ethnic groups in Yunnan Province and the surrounding areas, and 21 counties (cities) in Guizhou Province. More than 5300 accessions around Yunnan were collected, of which 89% were new, and more than 4800 accessions in Guizhou were collected, of which 73% were new. This work showed that there are many potential agricultural resources in ethnic minority areas of Yunnan and Guizhou. These germplasm resources were preserved and utilized primarily because of traditional ethnic culture and customs, reflected in the diet, festivals, weddings and funerals, decorations, medicine, farming culture and so on [68–70].

With the widespread popularization of hybrid rice and the impact of foreign culture, the number of varieties and cultivation area of KSR were decreasing. Although gene-bank conservation could stop the declines in the number of KSR varieties, the cultivation still decreased sharply. However, on-farm, in situ conservation was probably one of the most feasible, most economical and effective measures to conserve KSR. Based on the previous discussion, genetic variation of KSR influenced by many factors, including ethnic traditional culture and social customs, environment conditions, economic benefits, management measures and government policies, as well as farmers themselves, Dong farmers with different age, gender, and education level hold different perceptions to the cultivation and protection for KSR genetic resources. Therefore, in order to protect KSR, we should take some dramatic measures in relation to culture, environment, economy, management, policy, and farmers’ attitudes to carry out on-farm conservation. Firstly, traditional culture and social customs were the most important reasons for the conservation of KSR genetic resources. A large number of middle-aged people from Dong villages had been working outside since 2000, and they were increasingly unwilling to plant KSR. This change may be because the sense of identity with traditional culture had declined. In order to protect KSR resources and increase enthusiasm for planting KSR, it was necessary for the elders to actively guide these people to continue to grow KSR and increase the identity with ethnic traditional culture. In addition, the government should formulate laws and regulations to protect and promote the traditional culture of ethnic minorities. Secondly, local farmers should preserve KSR varieties suited to local climate and soil environment, including strong resistant to diseases and pests, drought, lodging, adapted to medium or low fertilizer soil, and high yield, discard unsuitable varieties. Related scientific research institutions and breeders should pay more attention to the collection, innovation, and utilization of KSR germplasm resources and promote Participatory Plant Breeding (PPB) programs that can provide a good basis for breeding high yield and good quality varieties. Thirdly, because of higher economic benefits of KSR and related by-products, local government should encourage and support farmers to establish KSR Cooperatives in cooperation with companies, expand KSR planting areas in large-scale and produce high quality of KSR rice, popularize “Rice-Fish-Duck Symbiotic Systems”, which could attain good social and economic benefits. Fourthly, KSR harvesting was time-consuming and laborious, which must be reaped by hand, if related machine tools were developed to instead of traditional handmade tools, it will promote the cultivation of KSR. Moreover, through the questionnaire survey of farmers’ attitudes to KSR, we found that a high level of education, female farmers, young and old farmers played more important roles in the cultivation and protection of KSR. Therefore, the government and related sections such as village and township committee should improve the education level of local farmers, promote the position of female farmers, and encourage the young people to cultivate KSR. All of these actions will protect the genetic diversity of KSR by the efforts of cooperatives, companies, and the government.

## Conclusions

KSR genetic resources were only distributed within the borders of Guizhou, Guangxi, and Hunan in China. Through the investigation in 33 ethnic villages, we found that although the number of varieties and the planting area of KSR had been greatly reduced in recent years, 156 KSR accessions had been still conserving by Dong farmers for thousands of years in the main producing areas—Congjiang, Rongjiang, and Liping counties of Guizhou Qiandongnan Prefecture. In addition, through the questionnaire survey, we found that ethnic traditional culture and social customs influenced the conservation of KSR, economic, management, and policy factors influenced the abandonment of KSR. By analyzing the correlation between farmers with different social characteristic and their attitude to the cultivation, reasons for conservation, reasons for abandonment, development tendency, and protection of KSR, we found that a high level of education, female, young, and old farmers play more important roles in the cultivation and protection of KSR. Therefore, we proposed improving the position of female farmers and the education level of young people and encouraging the old people to educate the middle-aged to conserve and protect KSR as well as Dong’s traditional culture and social customs. These suggestions will contribute to building on-farm conservation of KSR and preserving ethnic culture customs.

## Abbreviations

ABA: Abscisic Acid
BPH: Brown planthopper
FAO: The Food and Agriculture Organization
KSR: Kam Sweet Rice
PPB: Participatory Plant Breeding
